# Predictive testing for BRCA1/2: attributes, risk perception and management in a multi-centre clinical cohort

**DOI:** 10.1038/sj.bjc.6600253

**Published:** 2002-04-22

**Authors:** C Foster, D G R Evans, R Eeles, D Eccles, S Ashley, L Brooks, R Davidson, J Mackay, P J Morrison, M Watson

**Affiliations:** Psychology Research Group and Cancer Genetics Team, The Institute of Cancer Research, Sutton SM2 5PT, UK; Department of Medical Genetics, St Mary's Hospital, Manchester M13 0JH, UK; Department of Psychological Medicine and Cancer Genetics Team, Royal Marsden NHS Trust, London and Sutton SM2 5PT, UK; Wessex Clinical Genetics Service, Princess Ann Hospital, Southampton SO16 5YA, UK; Institute of Medical Genetics, Yorkhill NHS Trust, Glasgow G3 8SJ, Scotland, UK; Genetics Centre, Institute of Child Health, London WC1N, UK; Medical Genetics, Belfast City Hospital, Belfast BT9 7AB, Northern Ireland, UK

**Keywords:** predictive genetic testing, worry, risk management

## Abstract

The aim of this multi-centre UK study is to examine the attributes of a cohort offered predictive genetic testing for breast/ovarian cancer predisposition. Participants are adults unaffected with cancer from families with a known BRCA1/2 mutation. This is the first large multi-centre study of this population in the UK. The study evaluates mental health, perceived risk of developing cancer, preferred risk management options, and motivation for genetic testing. Participants were assessed when coming forward for genetic counselling prior to proceeding to genetic testing. Three hundred and twelve individuals, 76% of whom are female, from nine UK centres participated in the study. There are no gender differences in rates of psychiatric morbidity. Younger women (<50 years) are more worried about developing cancer than older women. Few women provide accurate figures for the population risk of breast (37%) or ovarian (6%) cancer but most think that they are at higher risk of developing breast (88%) and ovarian (69%) cancer than the average woman. Cancer related worry is not associated with perceived risk or uptake of risk management options except breast self-examination. The findings indicate that younger women may be particularly vulnerable at the time of the offer of a predictive genetic test.

*British Journal of Cancer* (2002) **86**, 1209–1216. DOI: 10.1038/sj/bjc/6600253 www.bjcancer.com

© 2002 Cancer Research UK

## 

Predictive genetic testing is available to some individuals unaffected with cancer where a BRCA1/2 mutation has been identified in the family (Miki *et al*, 1994; Wooster *et al*, 1995). Female gene mutation carriers not already affected by cancer have up to 85% and 27–60% chance of developing breast and ovarian cancer respectively in their lifetime (Ford *et al*, 1998). They are at higher risk of early onset (often pre-menopausal) and bilateral breast cancer compared to women at population risk (Ford *et al*, 1998). Male carriers have a slight increased risk of prostate and bowel cancer (Ford *et al*, 1994) and male BRCA2 carriers have an estimated 6% risk of developing breast cancer by the age of 70 years (Easton *et al*, 1997). The medical and psychological management of individuals eligible for predictive genetic testing must ensure that individuals are well prepared to enable them to make informed risk management decisions and minimise psychological distress experienced during the pre-test period.

Data from studies investigating predictive genetic testing for BRCA1/2 are relatively few at present. Most suggest that individuals taking up the offer of a predictive genetic test for BRCA1/2 are female (around 80%) and in their early to mid 40s (Lerman *et al*, 1997; Reichelt *et al*, 1999). Much of the literature focuses on highly researched individuals who participated in linkage studies (Croyle *et al*, 1997; Lerman *et al*, 1997). It is likely that important differences exist between individuals from ‘research’ families, where relatives are likely to be better informed and prepared for genetic testing, compared with those currently involved in testing. Adverse psychological effects of genetic testing may be more likely among individuals with less knowledge and risk awareness (Croyle *et al*, 1997). Levels of psychological morbidity and cancer specific worry in those presenting for genetic testing must be clarified to ensure they are well prepared, supported and able to cope with genetic test results.

### Men and hereditary breast and ovarian cancer

Men in families with hereditary breast/ovarian cancer (HBOC) can inherit a BRCA1/2 mutation and pass it on to their daughters and sons. DuDok de Wit *et al* (1996) described the psychological impact of HBOC on men in a small study of one family involved in linkage research. They suggest men may not come forward for genetic testing due to their low risk of developing cancer. In addition, minimisation and denial of the threat may lead men to avoid testing or fail to ask for support. Few studies have documented the attributes of men opting for genetic counselling and testing therefore a study including a substantial male cohort is needed.

### Mental health and cancer related worry

Women at high risk of HBOC may bear a heavy emotional burden due to their familial experiences of cancer, high bereavement rates and their own fears of developing the disease. Some women at increased risk of HBOC who have not yet been offered a genetic test experience high levels of distress (Lerman *et al*, 1993; Lloyd *et al*, 1996). Those proceeding with predictive genetic testing do not report higher levels of general distress (depression or anxiety) than women with one affected first-degree relative (Lerman *et al*, 1997) or population norms (Croyle *et al*, 1997). However, women have reported high levels of cancer specific distress prior to receiving a test result. Those most likely to report such distress at this time are young, more likely to anticipate problems if identified as a gene carrier, more likely to consider prophylactic mastectomy and more aware of the serious consequences of HBOC (Lodder *et al*, 1999). Cancer-related worry has been associated with increased interest in testing (Lerman *et al*, 1997).

### Risk perception

Women with HBOC tend to overestimate their risk of developing cancer despite genetic counselling (Evans *et al*, 1993; Watson *et al*, 1999). Cancer related distress (Lerman *et al*, 1995) and individual characteristics may interfere with comprehension of individualised genetic risk information. Research focusing on minority women from low-income households suggests that they are less likely to recognise breast cancer risk factors (Royak-Schaler *et al*, 1995). If genetic testing is to provide benefits it is important to ensure that those offered testing understand risk information and the advice given so that informed choices can be made in relation to risk management.

### Risk management

An important goal of predictive genetic testing is to help reduce mortality through early detection. There is limited evidence for the efficacy of mammography for pre-menopausal women. Recent evidence shows a benefit in women over 40 years (Duffy *et al*, 1996). Magnetic resonance imaging screening may be more sensitive in pre-menopausal women than mammography (Stoutjesdijk *et al*, 2001). Demographic variables such as age, education and ethnicity have been associated with differing levels of screening uptake (MacLean *et al*, 1984; Owens *et al*, 1987). Some women may opt for primary prevention strategies such as prophylactic surgery. The effectiveness of chemoprevention (e.g. tamoxifen) for female carriers of BRCA1/2 mutations is not yet known (Eeles and Powles, 2000).

### The role of anxiety and risk perception in risk management

Anxiety and misunderstanding of risk information may influence how individuals make use of risk management advice. Cancer related worry among relatives of breast cancer patients can interfere with adherence to breast screening recommendations (Alagna *et al*, 1987; Kash *et al*, 1992). However, the nature of the relationship between cancer worry and uptake of risk management options is unclear. Alagna *et al* (1987) report a curvilinear relationship between cancer worry and uptake of mammography where women with moderate levels of distress were more likely to engage in mammography than those with low or high levels.

Overestimation of risk is a potential barrier to screening. Kash *et al* (1995) found a negative correlation between perceived susceptibility and rate of breast self examination (BrSE), mammography and clinical breast examination (CBE: examination of breasts by a doctor) among women from families with HBOC. Different forms of risk management behaviour may be affected in different ways by cancer-related concerns and perceived risk.

### Reasons for predictive genetic testing

Reasons given by women undergoing BRCA1/2 testing include: obtaining certainty about carrier status; increasing the level of screening or to have prophylactic surgery; and learning about their children's risk (Lodder *et al*, 1999). An exploration of reasons for proceeding with genetic testing is needed within a cohort that is potentially less well informed than well-researched individuals and one that includes men.

The present study includes men and women from nine hospitals in England, Scotland and Northern Ireland that currently undertake the majority of BRCA1/2 testing in the UK. This cohort is representative of current clinical practice as few participants come from well-researched families. This is in contrast to much of the literature, which largely focuses on well-researched women. The study aim is to document mental health problems, clarify levels of cancer related distress, ascertain risk perceptions and examine ongoing and anticipated risk management behaviour in those offered genetic testing. Four key questions are addressed: (1) What are the levels of mental health problems and cancer related worry in a clinical cohort offered BRCA1/2 testing?; (2) Do women accurately estimate their risks of developing breast/ovarian cancer and are these related to cancer specific worry?; (3) What risk management behaviours do women engage in (or anticipate) and is this related to cancer worry and risk perception?; and (4) What is the motivation for predictive genetic testing and does this differ for men and women?

## METHODS

### Participants

Participants included 315 adults from nine UK clinical genetic centres. Those eligible were ⩾18 years, from families in which a BRCA1/2 mutation had been identified, had no previous diagnosis of cancer, and no current mental illness likely to be exacerbated by study participation.

### Procedure

This study was conducted as part of the baseline component of a large, prospective evaluation of BRCA1/2 testing in the UK. Eligible participants have a 50% (lower if an intervening relative has died) risk of inheriting a BRCA1/2 mutation. They are usually referred by their GP after learning of the mutation in the family. Some at risk individuals were directly informed of the availability of testing if they were already under the genetics service. They were recruited to the present study by their clinical geneticist or genetic associate/nurse between 1996 and 2000 during their clinic consultation (prior to the consultation at which blood was drawn for genetic analysis). Written informed consent was obtained. Participants were given a questionnaire to complete and return directly to the data management centre at the Institute of Cancer Research/Royal Marsden NHS Trust. The multi-centre research ethics committee (98Jan003) and all local research ethics committees approved the study.

### Measures

Measures were selected for validity, reliability and prior application to the study population along with a number of study-specific questions. The following pre-validated measures were included:

#### General Health Questionnaire

(GHQ28: Goldberg and Hillier 1979): a brief 28-item instrument designed to assess psychiatric disorder (cases) in non-psychiatric populations previously used with medical patients. The GHQ28 includes four sub-scales: somatic symptoms, anxiety and insomnia, social dysfunction, and severe depression. A total score on the GHQ28 (binary scoring) ranges from 0–28. A cut-off score of ⩾5 is recommended by the test authors i.e. a score ⩾5 indicates psychiatric disorder. However, Hopwood *et al* (1998) recommend a threshold of ⩾10 (binary scoring) for women with HBOC to reduce the overestimation of cases. We report the 10-point cut-off.

#### Cancer Worry Scale

(CWS: Lerman and Schwartz, 1993): this four-item scale assesses degree of worry about developing cancer (women only) using a 4-point Likert rating from ‘Not at all or rarely’ to ‘Almost all the time’. Two further items previously used elsewhere were included to ask about frequency of worry from ‘Not at all or rarely’ to ‘Constantly’ and how much of a problem this worry is from ‘Not at all’ to ‘Severe problem’ (Watson *et al*, 1999). This Revised 6-item Cancer Worry Scale (CWS-R) yields an internal reliability alpha coefficient of 0.87 (*n*=226 women). Maximum likelihood factor analysis explains 61% of the variance and a significant goodness of fit test χ^2^ (df=9)=25.75, *P*=0.003. A total score on the CWS-R ranges from 6–24. A high score indicates greater worry. No clinical cut-offs are currently available.

#### Impact of Event Scale

(IES: Horowitz *et al*, 1979): this scale determines levels of distress in response to a specific traumatic event. A modified 15-item version has previously been used to gather information on cancer-specific distress in high-risk and general population women (Kash *et al*, 1992; Watson *et al*, 1999). The IES was included to assess psychological response, with specific reference to thoughts about risk of cancer (women only) over the last 7 days. Total scores on the Intrusion and Avoidance scales range from 0–35 and 0–40 respectively. A high score indicates frequent intrusive/avoidant thoughts about risk of cancer.

#### Risk perception

Perceived risk of developing breast/ovarian cancer (women only) was assessed in terms of the likelihood of developing breast/ovarian cancer (3-point scale: ‘Not very likely’ to ‘Very likely’ or as a percentage or odds ratio) and relative risk compared to the average woman (5-point scale: ‘Very much lower’ to ‘Very much higher’). Knowledge of general population risk of breast/ovarian cancer was assessed by the question ‘Do you know what the average woman's risk is of breast/ovarian cancer throughout her lifetime?’ with response options ‘No’, ‘Yes – please specify’.

#### Risk management

Women were asked to indicate their current risk management practices and options they might consider should they be gene carriers. These included: mammography, chemo-prevention (participating in tamoxifen trial), prophylactic surgery, ultrasound, CBE, BrSE or any other screening for cancer. For BrSE women were asked about frequency. Barriers to screening were assessed using a 5-point scale from ‘Not difficult’ to ‘Very difficult’ including: physical discomfort, fear of examination, transport to screening clinic, distress caused by screening, and taking time from work/family/social obligations to attend (Kash *et al*, 1992).

#### Other measures

Study specific questions asked about reasons for wanting a genetic test (multiple choice) and whether participants considered themselves to be at increased risk of other cancers. Expectations about gene carrier status were assessed (‘certain I will not have the gene’, ‘uncertain’ and ‘certain I will have the gene’) and means of referral to the genetics clinic.

### Statistical methods

The association between categorical variables was examined by means of Fisher's exact test or the chi-squared test (χ^2^) with Yates correction. For ordered categorical variables the Mann–Whitney (MW) test for trend was used. Age was analysed as a continuous variable and, in addition, subjects were divided into three age groups (<35, 35–49, ⩾50 years). These three groups were chosen as risk management options vary for women in each group. Women under 35 years are unlikely to receive a mammogram and women over 50 years of age receive regular mammograms as part of the UK National Screening Programme.

Scores from the GHQ28, CWS-R and IES were treated as continuous variables. Normality was tested using the Kolmogorov-Smirnov statistic and parametric or non-parametric statistics were used as appropriate. Scores were summarised using mean (m) and standard deviation (s.d.) or median and range (in tables mean values have been quoted because of the relative invariance of the median). Groups were compared using analysis of variance or the Kruskal-Wallis (KW) test. Associations between psychological scores were summarised by the Pearson or Spearman correlation coefficients. The influence of a number of factors in predicting whether women practised BrSE was investigated in a multivariate logistic regression analysis. Subjects with missing data were omitted from the respective analyses.

## RESULTS

Three hundred and fifteen participants met study entry criteria; three declined (response rate 99%). Six participants were retrospectively identified as decliners of genetic testing (i.e. following their genetic counselling session) and therefore did not receive a questionnaire. Nine individuals did not return their questionnaire leaving a total of 298 (97%) completed questionnaires received (227 females; 71 males).

### Characteristics of the cohort

Participants at each centre were compared on demographic variables. Three centres (Manchester, London/Sutton and Southampton) accounted for 81% of the participants. Two of the smaller centres (Cambridge and Birmingham) only recruited women. There are no differences between the three larger centres (Manchester, London/Sutton and Southampton) except for London/Sutton patient's having a higher level of educational achievement than those from Southampton (*P*=0.02; MW) i.e. more of the London/Sutton participants attended college or university than the Southampton participants. Participants from the six smaller centres (*n*=57) are younger (*P*=0.001; MW) and have younger daughters (*P*=0.03; MW) than those from the three larger centres.

Seventy-six per cent of the cohort are women. Eighty-two per cent of participants are married or living with a partner. Over one third have a college or university education. Most men (75%) and women (66%) are currently employed. The median age for women is 41 (21–72 years), and 48 (22–86 years) for men. In the three age groups: 53 women and 14 men are <35 years; 120 women and 24 men 35–49 years; 54 women and 33 men ⩾50 years. Most participants have children (87%). The median age of offspring is 19 (range 0–50 years). Eighty-five per cent of participants described themselves as white.

### Type of referral

Participants were asked whose idea it was to attend the genetics clinic: 184 (63%) reported self-referral, 42 (14%) their family's idea, 12 (4%) GP recommendation and 31 (11%) referrals came from a genetics clinic. There were no significant gender differences in referral. Type of referral was not associated with general mental health (*P*=0.7; KW), cancer worry (*P*=0.9; KW), avoidant (*P*=0.7; KW) or intrusive (*P*=0.2; KW) thoughts, age (*P*=0.8; KW) or number of children (*P*=0.3; KW).

### General mental health and cancer related worry

The GHQ28, CWS-R and IES (Table 1

**Table 1:**
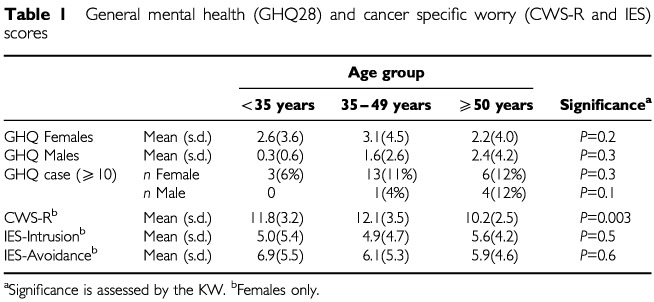
General mental health (GHQ28) and cancer specific worry (CWS-R and IES) scores

) assessed general mental health and cancer related concerns. Using a cut-off of ⩾10 (Hopwood *et al*, 1998) on the GHQ28, 22 (10%) females and five (7%) males met criteria for psychiatric disorder. Using the 5-point cut-off , 49 (22%) women and 10 (14%) men met the criteria for psychiatric disorder. There was no effect of gender or age on levels of psychiatric morbidity.

Younger women expressed higher levels of cancer worry (<50, median 12) than older women (⩾50, median 10) (*P*<0.001; MW). There was no difference between the two younger groups (<35 median 12; 35–49 median 11; *P*=0.9; MW). Forty-eight (21%) women stated that they worried about developing cancer ‘frequently’ or ‘constantly’ and 38 (17%) felt that their cancer-related worry was a ‘definite’ or ‘severe’ problem. Compared with older women, younger women worried more often (<50 years, 26% ⩾50 years, 7%; *P*=0.04 χ^2^) and found it more of a problem (<50 years, 21%; ⩾50 years, 4%; *P*=0.006 χ^2^). CWS-R and GHQ28 scores were positively correlated (0.4, *P*=0.01).

There were positive correlations (Pearson) between IES scales and GHQ28 (avoidance 0.4, *P*<0.01; intrusion 0.4, *P*<0.01) and CWS-R (avoidance 0.7, *P*<0.01; intrusion 0.5, *P*<0.01) scores. There was no effect of age on intrusion (*P*=0.5; KW) or avoidance (*P*=0.6; KW) scores. Fifty women (24% of the 205 women with an intrusion score) reported a total absence of intrusive thoughts (intrusion score=0). Thirty-six women (17% of the 207 with an avoidance score) recorded an avoidance score of 0.

### Risk perception

#### Own risk

Compared with the average woman, 197 (88%) and 153 (69%) thought they were at higher/much higher risk of developing breast and ovarian cancer respectively. Thirty (14%) and 70 (32%) women considered it not very likely that they would develop breast and ovarian cancer respectively. There was no relationship between perceived risk and GHQ28, CWS-R or IES scores. Self referred women (*n*=143) had higher perceived risk of breast cancer (not ovarian) with 97% of women reporting higher than average risk compared to 81% of the other referral groups (*n*=79; *P*=0.03; MW). Younger women (<50 years) have a higher perceived risk of breast (*P*=0.0005; MW) and ovarian (*P*=0.05; MW) cancer than older women.

#### Population risk

Sixty and 26% of women were able to give a figure for population risk of breast and ovarian cancer respectively. Of these, only 38% gave a correct estimate for breast (defined as odds ratio of 1 in 11–15) and 8% for ovarian cancer (defined as odds ratio of 1 in 50–100). Eighteen per cent and 16% of women overestimated the population risk of breast and ovarian cancer respectively. Only 4% and 1% of women underestimated population risk of breast and ovarian cancer respectively. Higher educational status was associated with accurate figures for population breast cancer risk. Forty-nine per cent of college/university educated participants were correct compared to 33% of those who were school educated (*P*=0.004; χ^2^). The corresponding figures for ovarian cancer were 14% college/university and 7% school educated (*P*=0.01). Age had no effect on accuracy of population ovarian cancer risk figures but younger women were more likely to give a correct figure for population breast cancer risk (<50 years, 43%; ⩾50 years, 23%; *P*=0.001; χ^2^).

### Risk of other cancers

Fifty-four (24%) women and 24 (34%) men felt that they were at increased risk of developing other cancers. Bowel (24 women, 13 men), prostate (12), lung (11 women, one man) and other gynaecological cancers (four) were the most common cancers mentioned.

### Risk management behaviour

Table 2

**Table 2:**
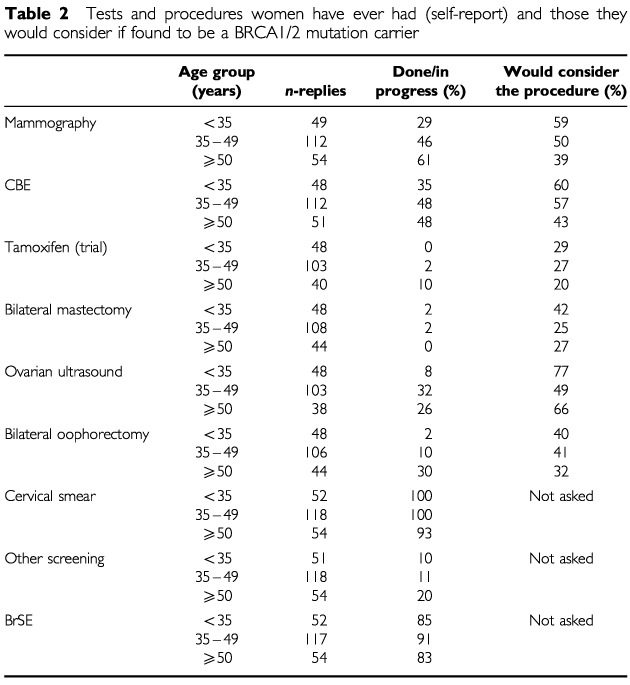
Tests and procedures women have ever had (self-report) and those they would consider if found to be a BRCA1/2 mutation carrier

illustrates the tests and procedures that women have ever had according to their self-reports and those that they may consider if they are found to be a gene carrier. This is shown according to age group. Risk management strategies were considered in relation to age and level of cancer-related worry.

#### Current risk management

These women reported having had a mammogram an average of 9 months previously (range 0 mn–10 years); a CBE an average of 7 months previously (range 0 mn–21 years). One woman reported having a bilateral mastectomy 2 years before (the other two do not report a date); ovarian ultrasound an average of 5 months previously (range 0 mn–7 years); and bilateral oophorectomy an average of 3 years previously (range 7 mn–9 years). One hundred and ninety-eight (88%) women reported performing BrSE; 22 (10%) more than once a week, 49 (22%) more than once a month, 68 (30%) monthly, 51 (22%) less frequently. Older women (⩾50 years) examined their breasts less frequently than the under 35 years (*P*=0.02) and the 35–49 groups (*P*<0.001; MW_Trend_). Two hundred and twenty-two (98%) women reported having had a cervical smear. Older women (⩾50 years) were less likely to report having had a cervical smear than younger women (*P*=0.001; χ^2^). Twenty-nine (13%) women reported other tests for cancer including biopsy (*n*=1), breast ultrasound (*n*=3), blood tests (*n*=2), CA125 (*n*=2), MRI (*n*=2), pelvic ultrasound (*n*=2), colonoscopy (*n*=4), and ‘other’ (not specified, *n*=7). These procedures were marginally more common in older women (⩾50 years; *P*=0.06; χ^2^).

Cancer-related worry was not associated with a higher level of risk management activity (i.e. women that had already had a procedure were not more worried than those who had not) other than for women performing BrSE. Women practising BrSE had a higher level of cancer related worry than those that did not (*P*=0.008; MW). A logistic regression analysis (adjusted for level of cancer worry) indicated that no other variables predicted BrSE where age, educational status, marital status, number of children, perception of the participants' own risk, their certainty of having the gene and other risk management strategies were entered as variables.

#### Anticipated risk management

If they were found to be a BRCA1/2 carrier younger women (<35 years) were more likely to consider prophylactic mastectomy than older women (⩾35 years; *P*=0.03; χ^2^). There was no relationship between cancer worry and anticipated risk management behaviour following the test result (MW). Comparing the number of women who had had the procedure, mammography was less common in younger women (<35 years; *P*=0.004; χ^2^).

### Barriers to screening

Women were asked to rate a number of factors that might make it difficult to attend for screening (Table 3

**Table 3:**
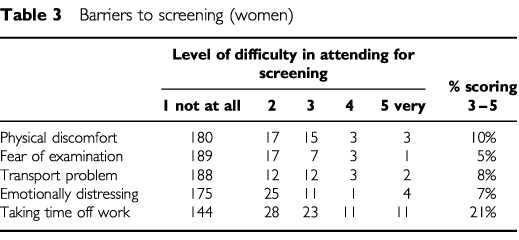
Barriers to screening (women)

). Taking time off work/family/social obligations was recorded as the factor causing most difficulty with 21% of women scoring 3–5. Older women were less likely to report taking time off work as a barrier (⩾50 years, 13%; <50 years, 40%; *P*=0.001; MW_Trend_). ‘Fear of the examination’ was more of a barrier to screening in the younger age group (<35 years, 25%; ⩾35 years, 9%; *P*=0.002; MW_Trend_). There were no other age differences.

### Reasons for testing

Reasons for wanting a genetic test are presented in Table 4

**Table 4:**
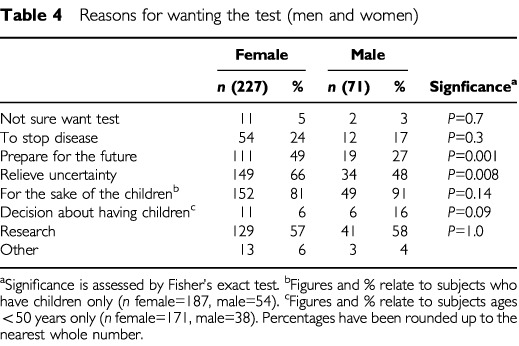
Reasons for wanting the test (men and women)

. Most women (81%) and men (91%) with children reported ‘for the sake of the children’. Mothers of daughters and older women were more likely to endorse ‘for the sake of the children’ than mothers of sons only (daughters 84%; sons 66%; *P*=0.02; Fisher) or younger women (⩾50 years, 83%; 35–49 years, 71%; <35 years, 52%; *P*=0.005; MW_Trend_). In the two younger groups, women under 35 years were more likely to endorse ‘to make decisions about having children’ (<35 years, 14%; 35–49 years, 3%; *P*=0.01; Fisher). Older women were less likely to give ‘to prepare for the future’ (⩾50 years, 31%; <50 years, 54%; *P*=0.005; Fisher). There were no other age differences between women. Women were more likely than men to want the test in order to prepare for the future (women, 49%; men, 27%; *P*=0.001; Fisher) and to relieve uncertainty (women, 66%; men, 48%; *P*=0.008; Fisher). Childless men and women were more likely to give ‘decisions about having children’ as a reason for wanting the test than those who were parents (women 67% *vs* 9%; *P*=0.001: men 67% *vs* 4%; *P*=0.007; Fisher). There was no relationship between psychiatric morbidity (GHQ) and reasons for wanting the genetic test.

When asked how certain they are about having a BRCA1/2 mutation the majority (75%) were uncertain, 22% were certain, and 3% certain that they did not. More women than men were certain (women, 25%; men, 12%; *P*= 0.03; Fisher).

## DISCUSSION

The aim of this multi-centre UK study is to examine the attributes of those who present for genetic testing for BRCA1/2. The most vulnerable group in this cohort is young women who report more cancer worry than older women. Worry about cancer is not associated with risk perception but it does impact on self-reported BrSE. Women who are more worried about developing cancer are most likely to perform BrSE.

### Mental health and cancer related worry

In this cohort women are more likely to report cancer specific distress than general psychological distress. This reflects findings from previous research (Kash *et al*, 1992; Croyle *et al*, 1997; Lerman *et al*, 1997). Younger women report more cancer specific worry than older women which is consistent with the literature (Lerman *et al*, 1993; Lloyd *et al*, 1996). A significant proportion of younger women (<50 years) worry more often about developing cancer and find it more of a problem than older women. In contrast, Lodder *et al* (1999) found that age was not associated with cancer specific distress although they did find that younger women had higher levels of general anxiety. In our cohort men and women do not report unusually high levels of general distress although these figures may be conservative given the raised GHQ28 threshold.

Most women in our study report some intrusive and avoidant thoughts about developing cancer and the numbers of women reporting no such thoughts are similar to Lodder *et al* (1999). However, the women in this cohort do not score as highly as those in Reichelt *et al* (1999) study although it is not clear from that study what they were asking women to rate on the IES. In our study women were asked to rate responses in relation to their risk of developing cancer. During genetic counselling prior to predictive genetic testing for BRCA1/2 it is important to ensure that decisions to proceed with genetic testing are informed and not motivated purely by cancer related worry. In this way impulsive decisions about testing that may be poorly informed can be avoided. Breast and ovarian cancer related concerns must be addressed prior to genetic testing. This is not to say that worry can or should be eliminated as concern about developing cancer given the family history is not surprising. However, it is important to ensure that appropriate services are in place to identify individuals most likely to need extra psychological support.

### Risk perception

To optimise health benefits individuals should understand risk information presented in genetic counselling so that informed decisions can be made regarding risk management. A significant proportion of women overestimate the population risk of breast and ovarian cancer. This is consistent with the literature (Evans *et al*, 1993; Watson *et al*, 1999). Younger women are more likely to provide an accurate figure for breast but not ovarian cancer risk. Most women in our study think it likely they will develop breast/ovarian cancer and that their risk is higher than the average woman. Self-referred women are more likely to think of themselves as at higher risk than those in the other referral groups. A higher perception of risk is related to the expectation of being a gene carrier. Lerman *et al* (1994) report that most women continue to overestimate their risk following genetic counselling, particularly those with high levels of concern about breast cancer. In this UK cohort cancer related worry was not associated with risk perception. This may be because the women in this study are eligible for predictive genetic testing and considering being tested and most of them consider themselves to be at increased risk. As such, given the low variation in risk perception scores it is difficult to assess the relationship between cancer worry and perception of risk at baseline.

### Risk management

In our study cancer related worry is positively associated with a higher level of BrSE but not with any other risk management option. In contrast to the literature (Alagna *et al*, 1987) we did not find a curvilinear relationship for mammography or other health behaviours. This discrepancy may be due to differences in the populations studied: in our study we included individuals at high risk eligible for predictive genetic testing whereas Alagna *et al*'s (1987) study included relatives of cancer patients who may not have been at risk themselves. We did not find a relationship between perceived risk and risk management behaviour as reported by Kash *et al* (1995). As previously stated this may be due in part to the low variation in perceived risk scores in our cohort. These differences may also be due to the age of our cohort. Younger women (<50 years) are more likely to report cancer related worry but are not necessarily eligible for mammography due to their age. In this cohort younger women are less likely to have had mammography than older women which reflects current screening guidelines. Women of all ages can perform BrSE therefore the relationship between health behaviour and cancer worry can be demonstrated.

With regards prophylactic surgery, more women have had and will consider prophylactic oophorectomy than mastectomy and younger women are more likely to consider prophylactic mastectomy than older women. In the older age group (⩾50 years) a substantial proportion of women have already had prophylactic oophorectomy. It is likely that prior to the availability of genetic testing prophylactic oophorectomy was chosen as a form of risk management in this group of high risk women in the absence of carrier status information. Actual uptake of risk management options following genetic testing will be established at follow-up. In this cohort cancer worry is not associated with consideration of prophylactic surgery. This is in contrast to other studies indicating that women with high levels of general anxiety (Lodder *et al*, 1999) and cancer specific worry (Stefanek, 1995) are more likely to consider prophylactic mastectomy. Women with HBOC who choose prophylactic mastectomy have been shown to be less anxious than those who do not (Hatcher *et al*, 2001). The number of women who indicated that they would consider this surgery was lower than that reported by Lodder *et al* (1999). While prophylactic surgery is still somewhat controversial, evidence suggests that risk is significantly reduced for women at high risk (Hartmann *et al*, 1999) and therefore offers hope that their risk of cancer will be reduced despite the presence of the BRCA1/2 mutation.

Given that one aim of predictive testing for BRCA1/2 is to reduce mortality by regular surveillance or surgery it is important to clarify concerns and support those having difficulty engaging in appropriate risk management behaviour. The most common barrier to screening reported by the women is taking time off from their usual obligations therefore practical steps can be taken to help women overcome such hurdles such as mobile mammography units that can be used by women under 50 years where appropriate.

### Motivation for testing

As with Lodder *et al* (1999) findings, the most commonly reported motivation for testing was for the sake of children. Females were more likely than men to want the test in order to prepare for the future and alleviate uncertainty. This indicates that women are thinking of their own risk as well as that of their children, unlike the men who are not at greatly increased risk. Traditional gene carrier testing and genetic counselling for late onset disease differ in that traditional genetic counselling typically focuses on risk to offspring while genetic counselling for susceptibility to breast/ovarian cancer involves personal risk information in addition to potential risk to offspring (Lerman *et al*, 1994). Younger women were more likely to give ‘to prepare for the future’ than older women. Childless men and women were more likely to give ‘decisions about having children’ than parents. At present prenatal carrier testing is not available for BRCA1/2 however it may become part of clinical practice in the future.

This study contributes to the psycho-oncology literature by investigating a large cohort including men and consisting mainly of individuals that have not come from well-researched families but from current clinical practice. We believe that this cohort represents a population of individuals now coming forward for BRCA1/2 testing who are new to this process. Most participants were recruited from three UK centres with established genetics services. Other centres were setting up their services during the recruitment phase of the study. Most participants were pre-menopausal women. This reflects findings from other studies (Lerman *et al*, 1997; Reichelt *et al*, 1999). Little is known about men coming forward for BRCA1/2 testing. Our sample includes a substantial group of men, most of whom are under 50 years of age and have children. Most men report initiating their referral to the genetics clinic, although relatives suggested a referral for a significant minority.

In summary, these data illustrate that while general mental health is not adversely affected by the prospect of predictive genetic testing, cancer related worry is particularly prevalent amongst pre-menopausal women many of whom self-refer to genetics services. In the future genetic testing is likely to become more rapid and more widely available. Given that many of the individuals in this cohort experience frequent and troublesome cancer worry and perceive themselves to be at high risk it is important that they continue to receive genetic counselling prior to testing in order to address concerns, receive information to make informed choices and continue to be supported both medically and psychologically.
